# Temporal patterns, incidence, and predictors of early stroke recurrence in atrial fibrillation

**DOI:** 10.1093/esj/23969873251352397

**Published:** 2026-01-01

**Authors:** Daniel Guisado-Alonso, Elisa Cuadrado-Godia, Ana Rodriguez-Campello, Isabel Fernández-Pérez, Adrià Macias-Gómez, Marta Vallverdú-Prats, Julia Peris-Subiza, Sergio Vidal-Notari, Laia Peraferrer-Montesinos, Jordi Jiménez-Conde, Joan Jiménez-Balado, Eva Giralt-Steinhauer, Angel Ois

**Affiliations:** Neurology Department, Hospital del Mar Research Institute, Barcelona, Spain; Neurology Department, Hospital del Mar Research Institute, Barcelona, Spain; Neurology Department, Hospital del Mar Research Institute, Barcelona, Spain; Neurology Department, Hospital del Mar Research Institute, Barcelona, Spain; Neurology Department, Hospital del Mar Research Institute, Barcelona, Spain; Neurology Department, Hospital del Mar Research Institute, Barcelona, Spain; Neurology Department, Hospital del Mar Research Institute, Barcelona, Spain; Neurology Department, Hospital del Mar Research Institute, Barcelona, Spain; Neurology Department, Hospital del Mar Research Institute, Barcelona, Spain; Neurology Department, Hospital del Mar Research Institute, Barcelona, Spain; Neurology Department, Hospital del Mar Research Institute, Barcelona, Spain; Neurology Department, Hospital del Mar Research Institute, Barcelona, Spain; Neurology Department, Hospital del Mar Research Institute, Barcelona, Spain

**Keywords:** Atrial fibrillation, stroke, recurrence, hemorrhage, anticoagulation

## Abstract

**Introduction:**

Early recurrence (ER) after an acute stroke event (ASE; ischemic or hemorrhagic) in patients with atrial fibrillation (AF) presents a therapeutic challenge due to the need to balance ischemic prevention with hemorrhagic risk. This study aimed to quantify ER incidence, both ischemic and hemorrhagic, and identify its predictors using real-world data from a prospective registry.

**Patients and methods:**

Retrospective analysis of patients with AF, either known or detected within 6 months, who were admitted for a first-ever ASE to a tertiary stroke center between 2005 and 2024. ER was defined as any recurrent event within 6 months. Baseline characteristics, CHA_2_DS_2_-VASc score, CHADS-VA score, stroke severity, anticoagulation type, AF detection timing, and monitoring duration were recorded. Cox and Fine-Gray models identified independent predictors.

**Results:**

Among 1795 patients, 108 (6.0%) experienced ER. The cumulative incidence was 6.3% (95% CI 5.1–7.4), and most events occurred within the first 30 days. Independent predictors included higher CHA_2_DS_2_-VASc score (sHR = 1.252, *p* = 0.023), lower initial stroke severity (sHR = 0.918, *p* < 0.001), concomitant stroke etiologies (sHR = 2.008, *p* = 0.001), and AF detected within 30 days after stroke (sHR = 1.644, *p* = 0.026). DOAC use was protective (sHR = 0.484, *p* = 0.003), while VKA showed a non-significant trend (sHR = 0.637, *p* = 0.068). Interaction analysis showed increased recurrence risk only in non-anticoagulated patients with AF detected after stroke. These findings were consistent across sensitivity analyses restricted to ischemic stroke, incorporating time-dependent anticoagulation, or comparing CHADS-VA and CHA_2_DS_2_-VASc scores.

**Conclusions:**

ER, predominantly ischemic, occurred mainly within 30 days. Risk factors included AF detection timing, CHA_2_DS_2_-VASc score, stroke severity, concomitant causes, and anticoagulation status, supporting early risk stratification and DOAC initiation.

## Graphical Abstract

**Graphical abstract:**
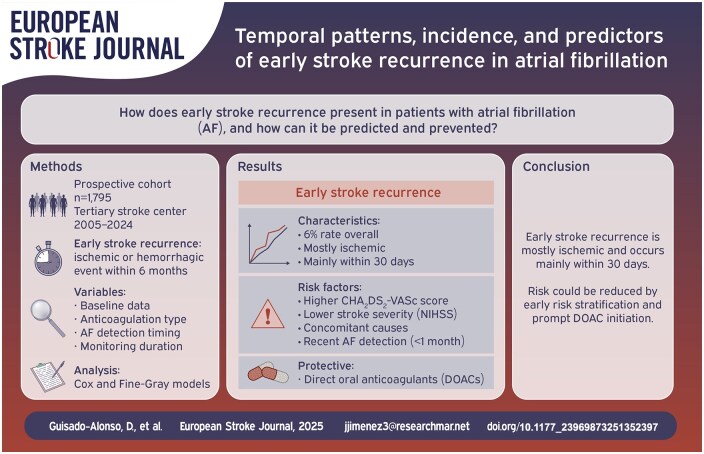

## Introduction

Early recurrence (ER), encompassing ischemic and hemorrhagic events after an acute stroke event (ASE) in patients with atrial fibrillation (AF) constitutes a significant therapeutic challenge due to the delicate balance between preventing ischemic recurrence with anticoagulation and mitigating hemorrhagic risk.^1–4^ Although anticoagulation reduces recurrence risk, trials evaluating direct oral anticoagulants (DOACs) in embolic stroke of undetermined sources (ESUS) have failed to show benefit.^5,6^ While prior studies provide important insights into the recurrence of ischemic and intracerebral hemorrhage (ICH) individually,^1–4^ comprehensive real-world data on ER, including both ischemic and hemorrhagic subtypes, remain limited.^3,7,8^ Both recurrence types significantly affect patient outcomes and therapeutic decisions, irrespective of their distinct pathophysiological mechanisms. We aimed to provide a detailed description of early-phase recurrence rates and identify predictors of ER in a large, prospective, hospital-based cohort of patients with AF who experienced an ASE.

## Methods

### Study population

**Figure 1.**
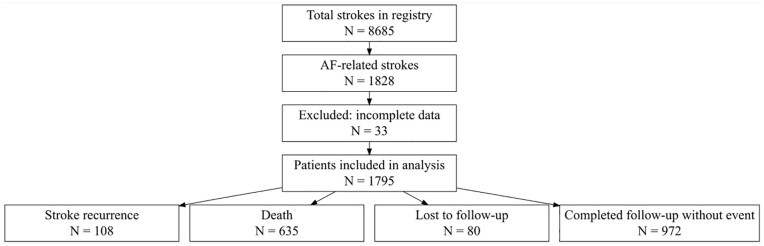
Patient inclusion flowchart. Among 8685 stroke patients, 1828 had atrial fibrillation (AF). After excluding 33 patients with incomplete data, 1795 were included in the final analysis. The figure provides a detailed breakdown of exclusions and final categorization of patients according to study outcomes.

We conducted a retrospective analysis based on a prospectively maintained registry of 8685 consecutive patients who experienced a first-ever ASE and were treated at a comprehensive tertiary stroke center between January 1, 2005, and June 1, 2024. For the present study, we selected 1828 patients with AF, either previously known or newly detected within 6 months of the index event. After excluding 33 patients due to incomplete data, a total of 1795 patients were included in the final analysis (see Figure 1).

### Data collection

We recorded demographic characteristics and vascular risk factors, including hypertension, diabetes mellitus, dyslipidemia, peripheral vasculopathy, coronary artery disease, heart failure, and valvulopathy. Lifestyle factors such as smoking and alcohol overuse were documented. Baseline functional status was assessed using the modified Rankin Scale, and initial stroke severity was measured using the National Institutes of Health Stroke Scale (NIHSS). Transient ischemic attack (TIA) was defined as a transient neurological deficit resolving within 24 h; TIA presentation referred to patients whose index event was a TIA. Post-discharge cardiac monitoring, hospital discharge decisions, anticoagulation therapy initiation and prolonged monitoring or additional testing were determined at the discretion of the treating vascular neurologist, guided by clinical parameters such as stroke severity, hemorrhagic risk, imaging findings, comorbidities; and in accordance with contemporary clinical guidelines/emerging evidence throughout the study period.^9–11^ As part of routine post-discharge management, anticoagulation therapy was initiated immediately upon AF detection in patients with AF detected after hospital discharge. Anticoagulation therapy was recorded during follow-up, including the exact date of initiation, and was coded as a binary variable (yes/no). For patients who received anticoagulation, treatment was also classified as Anticoagulant Type: vitamin K antagonist (VKA) or direct oral anticoagulant (DOAC). AF detection timing was categorized as: (1) known before stroke (including AF diagnosed before or at the time of stroke admission), (2) detected within 30 days, (3) detected between 30 and 90 days, or (4) detected after 90 days. To account for heterogeneity in post-discharge cardiac monitoring, monitoring duration after discharge was categorized into groups: Known at hospital discharge, ⩽3 days (short), >3 to <14 days (intermediate), and ⩾14 days (prolonged), including continuous or implantable devices. Ischemic Stroke etiology was classified using the Causative Classification of Stroke System (CCS: https://ccs.mgh.harvard.edu/), with cases involving additional competing factors categorized as concomitant stroke etiology (CSE).^12^ Stroke risk was assessed using the CHA_2_DS_2_-VASc score,^13,14^ which assigns points for HF, hypertension, age ⩾ 75 (2 points), DM, stroke/TIA/thromboembolism history (2 points), vascular disease, age 65–74 years, and sex category (female). Additionally, we calculated the CHADS_VA score,^15^ a simplified version excluding the sex category, to compare its predictive value with CHA_2_DS_2_-VASc.

### Study endpoint and follow-up

The primary endpoint was ER, defined as a new recurrent event – either ischemic or hemorrhagic – occurring within the first 6 months after the index ASE. The registry was prospectively designed to capture both ER and AF detection during this period, which was pre-specified for its clinical relevance and feasibility. The database included dates for ER, death, and loss to follow-up, enabling time-to-event analyses. Mortality data were also collected. Follow-up procedures included a clinical visit with a neurologist at 3 and 6 months post-ASE. All ER events, deaths, and hospital admissions were systematically reviewed using clinical records, neuroimaging, and hospital documentation. All ER events were adjudicated by senior stroke neurologists. In cases of diagnostic uncertainty, at least two senior stroke neurologists through joint review of clinical and/or imaging data reached a consensus.

### Statistical analyses

Baseline characteristics of the study cohort were summarized using median (interquartile range (IQR)) for continuous variables and frequencies (percentages) for categorical variables. Follow-up was censored at recurrence, death, or loss to follow-up, whichever occurred first. All patients were included in the time-to-event analysis. For descriptive purposes, we reported both the crude recurrence proportion (number of ER events divided by the total cohort size) and the cumulative incidence estimate derived from time-to-event analysis. The cumulative incidence accounted for censoring due to death or loss to follow-up, providing a more accurate risk estimate over time. To assess temporal patterns of stroke recurrence, follow-up time was divided into successive 30-day intervals up to 6 months. For each interval, person-time at risk was calculated by summing follow-up duration across all patients. Incidence rates for ischemic and hemorrhagic ER were expressed as events per person-year. Poisson regression models were used to estimate p-values, comparing incidence rates of each interval against the first month.

**Univariate analysis** was performed to compare patients with and without ER. For continuous variables, t-tests or Wilcoxon rank-sum tests were applied, depending on data distribution, while Chi-squared or Fisher’s exact tests were used for categorical variables. The corresponding *p*-values are presented in Table 1. The cumulative incidence function (CIF) was used to estimate ER risk, accounting for competing risks (mortality). Estimates with 95% confidence intervals (CI) were calculated. Since multiple comparisons were performed in Supplemental Table S1 (univariable Fine-Gray analyses), false discovery rate (FDR) adjustments were applied, and both *p*-values and *q*-values were reported. Among patients with AF detected after hospital admission, we calculated the recurrence rate prior to AF detection for each AF detection timing group (<1 month, 30–90 days, >90 days). This was an exploratory descriptive analysis. Recurrence rates prior to AF detection were calculated as crude proportions and were not adjusted for observation time. Comparisons between groups were performed using Fisher’s exact test.

**Table 1.** Baseline characteristics stratified by Early Stroke Recurrence (ER).

Variables	Total	No ER	ER	*p*-Value
	*N* = 1795	*N* = 1687	*N* = 108	
Age	82.0 [75.0;87.0]	82.0 [75.0;87.0]	81.0 [77.0;87.0]	0.991
CHA2DS2-VASc	6.00 [5.00;7.00]	6.00 [5.00;7.00]	7.00 [5.00;7.00]	**0.004**
CHADS-VA	5.00 [5.00;6.00]	5.00 [5.00;6.00]	6.00 [5.00;6.00]	**0.001**
Initial stroke severity (NIHSS)	8.00 [3.00;18.0]	8.00 [3.00;18.0]	4.00 [1.00;8.00]	**<0.001**
TIA presentation				**0.008**
No	1595 (88.9%)	1508 (89.4%)	87 (80.6%)	
Yes	200 (11.1%)	179 (10.6%)	21 (19.4%)	
Initial stroke type				0.155
Ischemic	1636 (91.1%)	1533 (90.9%)	103 (95.4%)	
Hemorrhagic	159 (8.86%)	154 (9.13%)	5 (4.63%)	
Previous Rankin Scale				0.073
0–2	1232 (68.6%)	1149 (68.1%)	83 (76.9%)	
3–5	563 (31.4%)	538 (31.9%)	25 (23.1%)	
Male gender				0.990
No	1071 (59.7%)	1006 (59.6%)	65 (60.2%)	
Yes	724 (40.3%)	681 (40.4%)	43 (39.8%)	
Hypertension				0.109
No	290 (16.2%)	279 (16.5%)	11 (10.2%)	
Yes	1505 (83.8%)	1408 (83.5%)	97 (89.8%)	
Diabetes mellitus				0.381
No	1175 (65.5%)	1109 (65.7%)	66 (61.1%)	
Yes	620 (34.5%)	578 (34.3%)	42 (38.9%)	
Dyslipidemia				0.107
No	941 (52.4%)	893 (52.9%)	48 (44.4%)	
Yes	854 (47.6%)	794 (47.1%)	60 (55.6%)	
Active smoker				0.944
No	1617 (90.1%)	1519 (90.0%)	98 (90.7%)	
Yes	178 (9.92%)	168 (9.96%)	10 (9.26%)	
Alcohol overuse				0.213
No	1673 (93.2%)	1576 (93.4%)	97 (89.8%)	
Yes	122 (6.80%)	111 (6.58%)	11 (10.2%)	
Concomitant causes				**<0.001**
No	1520 (84.7%)	1447 (85.8%)	73 (67.6%)	
Yes	275 (15.3%)	240 (14.2%)	35 (32.4%)	
Heart failure				0.112
No	1353 (75.4%)	1279 (75.8%)	74 (68.5%)	
Yes	442 (24.6%)	408 (24.2%)	34 (31.5%)	
Coronary disease				0.464
No	1531 (85.3%)	1442 (85.5%)	89 (82.4%)	
Yes	264 (14.7%)	245 (14.5%)	19 (17.6%)	
Peripheral vasculopathy				0.375
No	1645 (91.6%)	1549 (91.8%)	96 (88.9%)	
Yes	150 (8.36%)	138 (8.18%)	12 (11.1%)	
Valvulopathy				0.474
No	1592 (88.7%)	1499 (88.9%)	93 (86.1%)	
Yes	203 (11.3%)	188 (11.1%)	15 (13.9%)	
Anticoagulation therapy				0.948
No	701 (39.1%)	658 (39.0%)	43 (39.8%)	
Yes	1094 (60.9%)	1029 (61.0%)	65 (60.2%)	
Anticoagulants type				0.598
None	701 (39.1%)	658 (39.0%)	43 (39.8%)	
Vitamin K antagonist	473 (26.4%)	441 (26.1%)	32 (29.6%)	
Direct oral anticoagulant	621 (34.6%)	588 (34.9%)	33 (30.6%)	
AF detected timing				**0.007**
Known before stroke	1264 (70.4%)	1200 (71.1%)	64 (59.3%)	
<1 month	472 (26.3%)	436 (25.8%)	36 (33.3%)	
30–90 days	41 (2.28%)	34 (2.02%)	7 (6.48%)	
>90 days	18 (1.00%)	17 (1.01%)	1 (0.93%)	
AF monitoring duration (post-discharge)				**0.001**
Known at hospital discharge	1699 (94.7%)	1605 (95.1%)	94 (87.0%)	
Short (⩽3 days)	37 (2.06%)	29 (1.72%)	8 (7.41%)	
Intermediate (>3 to <14 days)	28 (1.56%)	27 (1.60%)	1 (0.93%)	
Prolonged (⩾14 days)	31 (1.73%)	26 (1.54%)	5 (4.63%)	

Baseline characteristics of the study population stratified by the presence or absence of early recurrence (ER) within 6 months. Continuous variables are presented as median [interquartile range]; categorical variables as count and percentage (*n*, %). CHA_2_DS_2_-VASc includes: congestive heart failure, hypertension, age ⩾ 75 years (2 points), diabetes mellitus, prior stroke/TIA/thromboembolism (2 points), vascular disease, age 65–74 years, and sex category (female). CHADS-VA is a simplified version of the CHA_2_DS_2_-VASc score that excludes sex category. Stroke severity was assessed using the National Institutes of Health Stroke Scale (NIHSS). Anticoagulation therapy indicates the use of oral anticoagulants at discharge or following atrial fibrillation (AF) detection after discharge. Anticoagulants type refers to whether patients received no anticoagulants, vitamin K antagonists (VKA), or direct oral anticoagulants (DOAC). AF detection timing classifies atrial fibrillation as known before stroke, or newly detected <1 month, between 30 and 90 days, or >90 days after stroke onset. AF monitoring duration (post-discharge) refers to the maximum duration of cardiac monitoring performed after hospital discharge and before 6 months. “None” indicates that no additional monitoring was done post-discharge (typically in patients with previously known AF). Bold *p*-values indicate statistical significance (*p* < 0.05).

### Multivariable models

To identify independent predictors of ER, we employed multivariable Cox regression models and Fine-Gray competing-risk models, with death treated as a competing event. Both models included baseline clinical factors such as initial stroke severity, CHA_2_DS_2_-VASc score, presence of CSE, anticoagulation therapy, AF detection timing, and monitoring duration after discharge. To avoid omitting clinically relevant factors, we applied a non-strict variable selection approach, incorporating all variables with clinical significance regardless of their individual statistical significance. Given potential collinearity between components of the CHA_2_DS_2_-VASc score and other covariates, we included only the total score to maintain model stability. We specifically assessed interaction terms between anticoagulation therapy (yes/no) and AF detection timing to evaluate whether anticoagulation modified the risk of recurrence depending on AF detection timing. This interaction term combined anticoagulation therapy (yes/no) and AF detection timing (known before stroke, <1 month, 30–90 days, >90 days). The multivariable Fine-Gray model included this interaction term and adjusted for baseline covariates. The results were visualized using a forest plot, displaying sub hazard ratios (sHR) and 95% CI. The reference group was anticoagulated patients with AF diagnosed before stroke. To compare the predictive performance of the CHA_2_DS_2_-VASc and CHADS_VA scores, we repeated both Cox and Fine-Gray models substituting one score for the other while retaining the same covariates. Model discrimination was assessed using Harrell’s concordance index (C-index), and model fit using the Akaike Information Criterion (AIC). Additional sensitivity analyses are presented in the Supplemental Material and detailed in Supplemental Tables S2–S4. All assumptions of the proportional hazards models were evaluated using Schoenfeld residuals and were globally satisfied (*p* > 0.05). Model fit and discrimination were evaluated using standard diagnostic plots and statistics. All statistical analyses were performed by a bioinformatician (J-J-B) using R statistical software (version 4.1.3, R Foundation for Statistical Computing). A two-tailed *p*-value < 0.05 was considered statistically significant.

### Ethics statement

The study was conducted in accordance with the Declaration of Helsinki and was approved by the local ethics committee. Written informed consent was obtained from all participants.

### Data availability

The data supporting the findings of this study are available from the corresponding author upon reasonable request.

## Results

### Study population and baseline characteristics

A total of 1795 patients were included in the analysis (Figure 1), comprising 1636 (91.1%) ischemic strokes and 159 (8.9%) ICH. Median follow-up was 5.98 months (IQR 5.38–6.00). Over the 6-month period, 80 patients (4.5%) were lost to follow-up and overall mortality reached 37.4% (95% CI 35.0–39.8). Baseline characteristics stratified by ER status are shown in Table 1. Patients with ER had higher CHA_2_DS_2_-VASc (7 vs 6, *p* = 0.004) and CHADS_VA scores (6 vs 5, *p* = 0.001), lower NIHSS (4 vs 8, *p* < 0.001), more frequent CSE (32.4% vs 14.2%, *p* < 0.001), and TIA presentation (19.4% vs 10.6%, *p* = 0.008). AF detection timing differed significantly across groups (*p* = 0.007), with early recurrence occurring in 64/1264 (5.06%) of patients with known AF before stroke, 36/472 (7.63%) for AF detected within 30 days, 7/41 (6.48%) for detection between 30 and 90 days, and 1/18 (5.56%) for detection after 90 days (Table 1). No significant differences were observed in anticoagulation status or anticoagulant type between groups.

### Incidence and temporal pattern of ER

Within 6 months, 108 of 1795 patients experienced early recurrence, corresponding to a crude recurrence rate of 6.0% (98 ischemic (5.5%), 10 hemorrhagic (0.6%)). The cumulative incidence estimate, adjusted for competing risks, was 6.3% (95% CI 5.1–7.4). The overall recurrence rate was 0.19 events per person-year, including 0.17 for ischemic and 0.02 for hemorrhagic events. Recurrence risk was numerically higher following ischemic stroke (103 of 1636; 6.3%) compared to ICH (5 of 159; 3.1%), although this difference did not reach statistical significance (*p* = 0.155). Recurrence rates across 30-day intervals (Table 2) were highest for ischemic events in the initial period (0.42 events per person-year), decreasing significantly over the following months (*p* < 0.001). Hemorrhagic recurrences remained infrequent across all intervals.

**Table 2.** Incidence rates of stroke recurrence by 30-day intervals.

Interval (months)	Recurrence (type)	Events	Person-time (days)	Person-time (years)	Incidence rate (events per person-year)	*p*-value (vs month 1)
1	Ischemic	49	42,334.75	115.98562	0.422	-
2		17	35,799.00	98.07945	0.173	0.002
3		8	33,454.00	91.65479	0.087	0.000
4		6	31,971.00	87.59178	0.068	0.000
5		7	30,744.00	84.23014	0.083	0.000
6		11	32,853.00	90.00822	0.122	0.000
1	Hemorrhagic	7	42,334.75	115.98562	0.060	-
2		0	35,799.00	98.07945	0.000	1.000
3		0	33,454.00	91.65479	0.000	1.000
4		2	31,971.00	87.59178	0.023	0.225
5		1	30,744.00	84.23014	0.012	0.128
6		0	32,853.00	90.00822	0.000	1.000

This table presents the incidence rates of ischemic and hemorrhagic stroke recurrence at successive 30-day intervals during the 6-month follow-up period. Events, person-time (in days and years), and incidence rates (per person-year) are provided separately for each recurrence type. *p*-Values are derived from Poisson regression models comparing each interval with the first month, within the same recurrence type.

### Univariable analysis

Cumulative incidence estimates and univariable predictors of ER are summarized in Supplemental Table S1. CHA_2_DS_2_-VASc (sHR 1.21, *p* = 0.012), CHADS_VA (sHR 1.26, *p* = 0.009), lower NIHSS (sHR 0.93, *p* < 0.001), CSE, and AF detected between 30 and 90 days were associated with higher ER risk. Recurrence rates were higher in patients with short (23%) or extended (16.8%) AF monitoring durations post-discharge, compared to intermediate durations (3.6%), with a significant overall difference across groups (*p* < 0.001). Predictors of mortality in the competing-risk analysis included older age, higher CHA_2_DS_2_-VASc and CHADS_VA scores, greater stroke severity, absence of anticoagulation, ICH subtype, and AF detection timing (Supplemental Table S1). Among patients with AF detected after hospital discharge (*n* = 531), ER rates prior to AF detection were higher in those with later detection (0.64% for <1 month, 7.3% for 30–90 days, 11.1% for >90 days; *p* = 0.00045; Supplemental Figure 1).

### Multivariable models

Multivariable Cox and Fine-Gray models are presented in Table 3. Given their consistency, we report Fine-Gray model results, which account for competing risks. Higher CHA_2_DS_2_-VASc (sHR = 1.252, 95% CI = 1.031–1.521, *p* = 0.023), lower NIHSS (sHR = 0.918, 95% CI = 0.89–0.946, *p* < 0.001), CSE (sHR = 2.008, 95% CI = 1.316–3.065, *p* = 0.001), and AF detected within 30 days after stroke (sHR = 1.639, 95% CI = 1.034–2.598, *p* = 0.035) remained significant predictors of ER. DOAC use was associated with reduced recurrence risk (sHR = 0.484, 95% CI = 0.298–0.786, *p* = 0.003), while VKA showed a non-significant trend (sHR = 0.637, 95% CI = 0.392–1.034, *p* = 0.068). AF monitoring duration after discharge and other clinical variables, including stroke type, heart failure, and coronary disease, showed no significant association with ER.

**Table 3.** Multivariable Cox and Fine-Gray models of early stroke recurrence in patients with atrial fibrillation.

Multivariable Cox model (full cohort)		*p*-Value
Variable	HR (95% CI)	
Stroke type (hemorrhagic vs. ischemic)	0.77 (0.29; 1.99)	0.5846
Initial stroke severity (NIHSS)	0.94 (0.91; 0.97)	<0.001
CHA_2_DS_2_-VASc score	1.28 (1.05; 1.55)	0.0128
Active smoker	1 (0.51; 1.98)	0.9887
Coronary artery disease	0.88 (0.51; 1.5)	0.6273
Heart failure	1.06 (0.64; 1.76)	0.8107
Valvulopathy	1.04 (0.58; 1.85)	0.9057
Concomitant stroke etiology	2.03 (1.33; 3.1)	0.0011
Anticoagulants type: VKA	0.5 (0.3; 0.81)	0.0052
Anticoagulants type: DOAC	0.37 (0.22; 0.6)	<0.001
AF monitoring duration: short (⩽3 days)	1.91 (0.69; 5.25)	0.2125
AF monitoring duration: intermediate (>3 to <14 days)	0.42 (0.05; 3.67)	0.4365
AF monitoring duration: prolonged (⩾14 days)	2.49 (0.82; 7.6)	0.1085
AF detected timing: <1 month	1.61 (1.03; 2.51)	0.0359
AF detected timing: 30–90 days	2.18 (0.66; 7.23)	0.2013
AF detected timing: >90 days	0.58 (0.07; 5.17)	0.6264
Fine-Gray model (full cohort)		
Variable	β (95% CI)	
Stroke type (hemorrhagic vs. ischemic)	0.722 (0.285–1.827)	0.492
Initial stroke severity (NIHSS)	0.918 (0.89–0.946)	<0.001
CHA_2_DS_2_-VASc score	1.252 (1.031–1.521)	0.023
Active smoker	0.993 (0.51–1.934)	0.983
Coronary artery disease	0.889 (0.513–1.541)	0.675
Heart failure	1.045 (0.625–1.747)	0.866
Valvulopathy	1.094 (0.611–1.958)	0.762
Concomitant stroke etiology	2.008 (1.316–3.065)	0.001
Anticoagulants type: VKA	0.637 (0.392–1.034)	0.068
Anticoagulants type: DOAC	0.484 (0.298–0.786)	0.003
AF monitoring duration: short (⩽3 days)	2.245 (0.831–6.067)	0.111
AF monitoring duration: intermediate (>3 to <14 days)	0.444 (0.058–3.428)	0.437
AF monitoring duration: prolonged (⩾14 days)	2.419 (0.853–6.857)	0.097
AF detected timing: <1 month	1.644 (1.061–2.546)	0.026
AF detected timing: 30–90 days	2.12 (0.717–6.264)	0.174
AF detected timing: >90 days	0.591 (0.058–6.022)	0.657
Fine-Gray model with interaction: anticoagulation × AF detection		
Variable	sHR (95% CI)	
Stroke type (hemorrhagic vs. ischemic)	0.772 (0.306–1.951)	0.585
Initial stroke severity (NIHSS)	0.918 (0.89–0.947)	<0.001
CHA_2_DS_2_-VASc score	1.262 (1.039–1.531)	0.019
Active smoker	0.995 (0.511–1.937)	0.988
Coronary artery disease	0.906 (0.523–1.568)	0.724
Heart failure	1.037 (0.622–1.731)	0.888
Valvulopathy	1.145 (0.65–2.017)	0.638
Concomitant stroke etiology	1.964 (1.279–3.014)	0.002
AF monitoring duration: short (⩽3 days)	1.993 (0.702–5.655)	0.195
AF monitoring duration: intermediate (>3 to <14 days)	0.537 (0.071–4.072)	0.547
AF monitoring duration: prolonged (⩾14 days)	2.633 (0.934–7.42)	0.067
Interaction: AC + Known AF (ref)	0.664 (0.394–1.12)	0.125
Interaction: No AC + AF < 1 month	2.144 (1.141–4.03)	0.018
Interaction: AC + AF < 1 month	0.944 (0.49–1.817)	0.863
Interaction: No AC + AF 30–90 days	4.78 (1.117–20.454)	0.035
Interaction: AC + AF 30–90 days	0.945 (0.26–3.433)	0.931
Interaction: No AC + AF > 90 days	0 (0–0.001)	<0.001
Interaction: AC + AF > 90 days	0.568 (0.058–5.531)	0.626

AC: anticoagulation; AF: atrial fibrillation; CI: confidence interval; DOAC: direct oral anticoagulant; NIHSS: National Institutes of Health Stroke Scale; sHR: subdistribution hazard ratio; VKA: vitamin K antagonist.

Multivariable Fine-Gray competing risk model assessing predictors of early stroke recurrence in patients with atrial fibrillation (AF). The model includes an interaction between anticoagulation therapy (AC) and timing of AF detection (known before stroke, <1 month, 30–90 days, >90 days). AF monitoring duration after discharge was categorized as: short (⩽3 days), intermediate (>3 to <14 days), or extended (⩾14 days, including continuous or implantable monitoring). The reference group for the interaction was anticoagulated patients with a known diagnosis of AF prior to hospital discharge. Subdistribution hazard ratios (sHR), 95% confidence intervals (CI), and p-values are reported. Death was treated as a competing event.

### Interaction between anticoagulation and AF detection timing

Including the interaction term between anticoagulation and AF detection timing in the Fine-Gray competing-risk model demonstrated that ER risk was significantly higher in non-anticoagulated patients with AF detected within < 1 month (sHR = 2.14, 95% CI = 1.14–4.03, *p* = 0.018) and between 30 and 90 days (sHR = 4.78, 95% CI = 1.12–20.45, *p* = 0.035), whereas no recurrences occurred in non-anticoagulated patients with AF detected >90 days (*p* < 0.001), when compared to anticoagulated patients with known AF before stroke (reference group). In anticoagulated patients, AF detection timing was not associated with ER (all *p* > 0.05). These findings, adjusted for CHA_2_DS_2_-VASc, NIHSS, CSE, and other covariates, are presented in Supplemental Figure 2.

## Discussion

This study provides comprehensive insights into the incidence, temporal patterns, and predictors of ER after ASE in patients with AF, based on a large real-world cohort. In multivariable models, higher CHA_2_DS_2_-VASc, lower NIHSS, CSE, AF detection timing, and anticoagulant type were identified as independent predictors of recurrence risk. Recurrence risk after ischemic SAE was predominantly ischemic rather than ICH, a finding consistent with previous literature range from 3.0% to 7.6%.^1–3,16^ ER following an ICH was 3.1%; however, these rates remain less well known, ranging from 3.2% to 12.7% for ischemic events and from 1.5% to 3.9% for recurrent ICH events.^3,17,18^

### Temporal pattern and clinical implications

In our cohort, ER occurred predominantly as ischemic events, particularly following an ischemic ASE, reflecting both the underlying embolic risk of AF and treatment strategies favoring earlier anticoagulation initiation in this group. Although less frequent after ICH, a measurable risk of ischemic recurrence persisted (5 of 159; 3.1%), consistent with prior reports.^3,17,18^ This difference in recurrence patterns, though not statistically significant, likely reflects both pathophysiological mechanisms and clinical decision-making balancing hemorrhagic and thromboembolic risks. The significantly elevated ER risk during the first month post-stroke highlights a critical window for intervention. This pattern is consistent with previous studies showing that ischemic events in AF-related stroke are most frequent during the first weeks, although many of those studies used broader time intervals or excluded patients with early mortality, severe strokes, or hemorrhagic events.^1–3,16,19^ In contrast, by including all patients regardless of recurrence type or clinical complexity, our cohort offers a more representative estimation of recurrence risk over time, better reflecting real-world conditions. These findings highlight the need for early risk stratification and individualized prevention strategies during the acute to early subacute phases, where the potential for improving outcomes is greatest in this high-risk population. Additionally, the high overall mortality observed within the first 6 months reflects the advanced age and clinical complexity of patients with AF-related cardioembolic stroke, consistent with prior reports.^3,18–20^

### Predictors of early recurrence

CSE was a consistent and independent predictor of ER across all models, underscoring its impact during the early post-stroke period. A strength of our study is the systematic and comprehensive etiological evaluation conducted in all patients, which enabled accurate identification of coexisting stroke mechanisms – such as atherosclerosis – that likely contribute to the elevated long-term recurrence risk associated with CSE.^12,21^ Our finding is consistent with the well-established risk of ER observed in atherothrombotic strokes.^22^ Although CSE and the CHA_2_DS_2_-VASc score capture different dimensions of vascular risk, both share common clinical components, including hypertension and diabetes.^13,23,24^ In our cohort, CHA_2_DS_2_-VASc remained independently associated with ER, reinforcing its prognostic value in AF patients. Given that sex (female) did not associate with recurrence risk in our analyses, we compared CHA_2_DS_2_-VASc with the simplified CHADS_VA score,^15^ which excludes sex as a component. Both scores demonstrated similar predictive performance in multivariable models. These findings support the clinical utility of CHADS_VA as a practical alternative for risk stratification, consistent with recent external validations.^1,15^ Initial stroke severity was inversely associated with ER in our cohort. However, the relationship between stroke severity and ER is complex and remains unclear in the literature, with inconsistent findings across observational studies depending on stroke subtype, anticoagulation strategies, follow-up duration, and statistical approach.^1–3,7,19,20^ Although early mortality could contribute to this pattern by precluding recurrence events in patients with severe strokes, our results remained significant in the Fine–Gray competing-risk model, which accounts for death as a competing event. This reinforces the robustness of the association. Clinically, these findings highlight the importance of early secondary prevention, including the potential value of ultra-early and prolonged cardiac monitoring in patients with mild neurological deficits, who may otherwise be considered at lower risk. The role of stroke severity in recurrence risk stratification remains unresolved and warrants further investigation. TIA presentation, although associated with recurrence in univariable analysis, was excluded from multivariable models due to collinearity with NIHSS. Stroke type (ischemic vs ICH) was not independently associated with ER in adjusted models, suggesting that once clinical covariates and treatment strategies are accounted for, index event type does not exert an independent effect on recurrence risk. To further evaluate these predictors in a more uniform pathophysiological context, we conducted a sensitivity analysis restricted to patients with ischemic stroke as the index event and ischemic recurrences as the outcome (Supplemental Table S2). This analysis confirmed the consistency of the primary predictors – CHA_2_DS_2_-VASc, NIHSS, CSE, and anticoagulation therapy – supporting the robustness of our findings despite the inclusion of both ischemic and hemorrhagic events in the main analysis.

### AF detection timing, cardiac monitoring, and anticoagulation

Finally, the interplay between AF detection timing, arrhythmic burden, cardiac monitoring strategies, and anticoagulation therapy remains complex, particularly given the well-known lack of efficacy of empiric anticoagulation in cryptogenic stroke patients without confirmed AF, as demonstrated in randomized trials.^5^ This underscores the need for precise identification of patients at highest thromboembolic risk.^1,4^ Our analysis showed that a small but clinically relevant proportion of patients experienced ER prior to AF detection, with increasing frequency in later detection groups (see Supplemental Figure 1). This may suggest that some ER occur during a period of undiagnosed AF, reinforcing the concept of a vulnerable window after stroke. However, these proportions were not adjusted for differing observation times across groups, and the higher rates observed with later AF detection may partly reflect longer periods at risk before diagnosis. The low absolute number of such cases helps explain why empiric anticoagulation for all, as done in ESUS trials, has not proven effective^5^: the risk is concentrated in a minority. These findings support targeted and individualized approaches and may also support the hypothesis that ultra-early and continuous cardiac monitoring could reduce the likelihood of stroke recurrence during periods of undetected AF in selected patients.^1,25^

In our cohort, AF detected within the first 90 days post-stroke, particularly within the first month, was associated with higher recurrence rates, suggesting a subgroup with greater thromboembolic potential. While previous studies have shown that patients with previously known AF tend to exhibit higher long-term recurrence risk,^2,4,26^ our findings suggest that early-detected AF, particularly within the first month post-stroke, is associated with the highest ER risk in the absence of anticoagulation. This may reflect a combination of factors, including differences in stroke severity, intensity of cardiac monitoring during the acute phase, treatment decisions, and the arrhythmic burden of newly detected AF episodes.^1,4,7,26^ Importantly, the association between early AF detection and higher recurrence risk was observed only in non-anticoagulated patients, as shown in an interaction analysis using a Fine–Gray competing-risk model (Supplemental Table S2), with anticoagulated patients with known AF before stroke as the reference group. In anticoagulated individuals, no significant association was observed between recurrence risk and AF detection timing. AF detected beyond 90 days showed limited recurrence potential, although the small size of this subgroup warrants cautious interpretation. Later-detected AF may reflect lower arrhythmic burden and reduced thromboembolic risk, as suggested by previous studies,^4,26^ which aligns with our observations. Monitoring duration was included in all multivariable models, but no significant association with ER was observed. While prolonged monitoring may detect AF with lower burden and reduced thromboembolic risk, this hypothesis could not be fully assessed in our real-world cohort, where monitoring strategies varied according to clinical judgment and guideline evolution.^4^ Recognizing the dynamic nature of anticoagulation exposure, we further refined our analysis using a Fine-Gray model with anticoagulation as a time-dependent covariate (Supplemental Table S4). This approach accounted for the timing of anticoagulation initiation relative to recurrence events, providing a more accurate estimation of its protective effect. The time-dependent model confirmed that anticoagulation consistently reduced recurrence risk, independent of CHA_2_DS_2_-VASc, NIHSS, CSE, and AF detection timing. These findings emphasize the importance not only of anticoagulation use but also of its timely initiation relative to recurrence risk. This supports prompt anticoagulation initiation once clinically feasible, particularly in patients with early AF detection post-stroke, and aligns with emerging evidence favoring earlier anticoagulation resumption to balance thromboembolic prevention and hemorrhagic risk more effectively.

### Strengths and limitations

This study’s strengths include the large, clinically diverse cohort, real-world data collection, systematic etiological evaluation, and robust statistical approaches incorporating competing-risk models. Sensitivity analyses – such as the restriction to ischemic index events, adjustment for anticoagulant type, and the head-to-head comparison between CHA_2_DS_2_-VASc and CHADS_VA scores – enhance methodological rigor and address specific reviewer concerns. Limitations include the retrospective design and the evolving treatment standards over the extended recruitment period, which may introduce residual confounding. In addition, heterogeneity in post-discharge cardiac monitoring – driven by clinical discretion and changing guideline recommendations – represents a methodological limitation. Small subgroup sizes, particularly in cases of late-detected AF and hemorrhagic recurrence, limit the statistical power of some analyses. Moreover, no formal sample size calculation was performed, as this was a retrospective analysis of a predefined registry cohort.

### Conclusion

In summary, ER after stroke in patients with AF is predominantly ischemic and occurs mainly in the early post-stroke period. The main predictors were CHA_2_DS_2_-VASc or CHADS_VA scores, initial stroke severity, CSE, AF detection timing, and anticoagulant therapy. The time-dependent analysis of anticoagulation exposure highlights the importance of both initiating therapy and its timing in reducing recurrence risk. These findings provide robust evidence to guide clinical decision-making and support further research to refine secondary prevention strategies.

## Supplementary Material

sj-docx-1-eso-23969873251352397
