# Supplementary material for: Temporal patterns, incidence, and predictors of early stroke recurrence in atrial fibrillation
Source: Eur Stroke J. 2026 Jan 1;11(1):23969873251352397. doi: 10.1093/esj/23969873251352397 (PMC12866207; doi:10.1093/esj/23969873251352397)
Supplement: sj-docx-1-eso-23969873251352397 [file sj-docx-1-eso-23969873251352397.docx]

**Statistical Analyses**

**Sensitivity analyses**
We conducted a separate Fine-Gray competing-risk model restricted to patients with ischemic stroke as the index event, considering only ischemic stroke recurrence as the outcome. The model structure and covariates remained unchanged (see Supplementary Table S2). Additionally, we performed a Fine-Gray competing-risk model incorporating anticoagulation therapy as a time-dependent covariate to dynamically assess its effect on ER risk over the follow-up period. This approach allows for more accurate modeling of anticoagulation exposure, adjusting for baseline covariates, AF detection timing, and monitoring duration (Supplementary Table S4).

**Sensitivity analyses**

A Fine-Gray competing-risk model restricted to patients with ischemic stroke as the index event confirmed the robustness of the primary findings (Supplementary Table S2). In this model, higher CHA₂DS₂-VASc (sHR=1.294, 95% CI=1.054–1.588, p=0.014), lower NIHSS (sHR=0.919, 95% CI=0.888–0.952, p<0.001), CSE (sHR=1.979, 95% CI=1.27–3.084, p=0.003), and AF monitoring duration ≥14 days (sHR=3.357, 95% CI=1.156–9.746, p=0.026) remained significant predictors of ER. The interaction between anticoagulation therapy and AF detection timing remained consistent, with higher recurrence risk observed in non-anticoagulated patients with AF detected within 30 days after stroke (sHR=2.236, 95% CI=1.123–4.45, p=0.022) or between 30–90 days (sHR=4.763, 95% CI=1.101–20.593, p=0.037), and no recurrences in those with AF detected >90 days without anticoagulation (p<0.001). Additionally, a Fine-Gray competing-risk model incorporating anticoagulation therapy as a time-dependent covariate demonstrated its protective effect against ER (sHR=0.371, 95% CI=0.249–0.551, p<0.001; Supplementary Table S4). In this model, higher CHA₂DS₂-VASc (sHR=1.346, 95% CI=1.1–1.646, p=0.004), lower NIHSS (sHR=0.962, 95% CI=0.931–0.994, p=0.020), and CSE (sHR=2.213, 95% CI=1.431–3.424, p<0.001) remained significant predictors of ER, while AF detection timing and monitoring duration did not show significant associations. A separate analysis comparing CHA₂DS₂-VASc and CHADS_VA scores in multivariable models (Supplementary Table S3) showed similar predictive performance. Both scores remained independently associated with ER in Fine-Gray models (CHA₂DS₂-VASc: sHR=1.252, 95% CI=1.031–1.521, p=0.023; CHADS_VA: sHR=1.27, 95% CI=1.001–1.612, p=0.049). In Cox models, discrimination and model fit were comparable between scores (C-index: 0.711 vs 0.707; AIC: 1509.3 vs 1509.5).

**Supplementary Table S1.** **Univariable Fine-Gray competing-risk analysis of clinical predictors for early stroke recurrence, considering death as a competing event**

| **Outcome** | **Variable** | **Group** | **Measure** | **p-val** | **Q-val** |
| --- | --- | --- | --- | --- | --- |
| Study Event | Age |  | 1 (0.99; 1.02) | 0.650 | 0.650 |
|  | Initial stroke severity (NIHSS) |  | 0.93 (0.9; 0.95) | p < 0.001 | 0.002 |
|  | CHA₂DS₂-VASc |  | 1.21 (1.04; 1.4) | 0.012 | 0.016 |
|  | CHADS-VA |  | 1.26 (1.06; 1.5) | 0.009 | 0.016 |
|  | TIA presentation | No | 5.7% (4.6%; 6.9%) | 0.004 | 0.024 |
|  |  | Yes | 10.9% (6.5%; 15.3%) |  |  |
|  | Initial stroke type | Ischemic | 6.6% (5.4%; 7.9%) | 0.091 | 0.182 |
|  |  | Hemorrhagic | 3.1% (0.4%; 5.9%) |  |  |
|  | Previous Rankin Scale | 0-2 | 7.1% (5.6%; 8.6%) | 0.046 | 0.166 |
|  |  | 3-5 | 4.6% (2.8%; 6.3%) |  |  |
|  | Male gender | No | 6.4% (4.9%; 7.9%) | 0.857 | 0.885 |
|  |  | Yes | 6.2% (4.4%; 8%) |  |  |
|  | Hypertension | No | 4.1% (1.7%; 6.6%) | 0.072 | 0.182 |
|  |  | Yes | 6.7% (5.4%; 8%) |  |  |
|  | Diabetes mellitus | No | 5.9% (4.5%; 7.3%) | 0.332 | 0.498 |
|  |  | Yes | 7.1% (5%; 9.2%) |  |  |
|  | Dyslipidemia | No | 5.3% (3.9%; 6.8%) | 0.077 | 0.182 |
|  |  | Yes | 7.4% (5.6%; 9.2%) |  |  |
|  | Active smoker | No | 6.3% (5.1%; 7.5%) | 0.768 | 0.864 |
|  |  | Yes | 6.1% (2.4%; 9.8%) |  |  |
|  | Alcohol overuse | No | 6.1% (4.9%; 7.3%) | 0.154 | 0.277 |
|  |  | Yes | 9.3% (4.1%; 14.6%) |  |  |
|  | Concomitant causes (CSE) | No | 5.1% (3.9%; 6.2%) | p < 0.001 | p < 0.001 |
|  |  | Yes | 13.4% (9.2%; 17.5%) |  |  |
|  | Heart failure | No | 5.8% (4.5%; 7.1%) | 0.087 | 0.182 |
|  |  | Yes | 7.9% (5.4%; 10.5%) |  |  |
|  | Coronary disease | No | 6.1% (4.9%; 7.3%) | 0.367 | 0.508 |
|  |  | Yes | 7.5% (4.3%; 10.8%) |  |  |
|  | Peripheral vasculopathy | No | 6.1% (4.9%; 7.3%) | 0.284 | 0.465 |
|  |  | Yes | 8.4% (3.8%; 12.9%) |  |  |
|  | Valvulopathy | No | 6.1% (4.9%; 7.4%) | 0.442 | 0.568 |
|  |  | Yes | 7.5% (3.9%; 11.2%) |  |  |
|  | Anticoagulation therapy | No | 6.5% (4.6%; 8.3%) | 0.885 | 0.885 |
|  |  | Yes | 6.2% (4.8%; 7.7%) |  |  |
|  | Anticoagulants type | No anticoagulation | 6.5% (4.6%; 8.3%) | 0.530 | 0.636 |
|  |  | Vitamin K antagonist (VKA) | 7.1% (4.7%; 9.5%) |  |  |
|  |  | Direct oral anticoagulant (DOAC) | 5.6% (3.7%; 7.4%) |  |  |
|  | AF detection timing | known before stroke | 5.2% (4%; 6.5%) | 0.005 | 0.025 |
|  |  | <1 Month | 8.1% (5.6%; 10.7%) |  |  |
|  |  | 30-90 Days | 18.3% (5.6%; 31.1%) |  |  |
|  |  | >90 Days | 5.6% (0%; 16.4%) |  |  |
|  | AF monitoring duration (post-discharge) | Known at Hospital Discharge | 5.7% (4.6%; 6.9%) |  |  |
|  |  | Short | 23% (8.5%; 37.4%) |  |  |
|  |  | Intermediate | 3.6% (0%; 10.6%) |  |  |
|  |  | Extended | 16.8% (3%; 30.5%) | p < 0.001 | p < 0.001 |
| Competing Event | Age |  | 1.06 (1.05; 1.07) | p < 0.001 | p < 0.001 |
|  | Initial stroke severity (NIHSS) |  | 1.11 (1.1; 1.13) | p < 0.001 | p < 0.001 |
|  | CHA₂DS₂-VASc |  | 1.26 (1.19; 1.34) | p < 0.001 | p < 0.001 |
|  | CHADS-VA |  | 1.29 (1.21; 1.37) | p < 0.001 | p < 0.001 |
|  | TIA presentation | No | 40% (37.5%; 42.5%) | p < 0.001 | p < 0.001 |
|  |  | Yes | 7.1% (3.3%; 10.9%) |  |  |
|  | Initial stroke type | Ischemic | 34% (31.7%; 36.4%) | p < 0.001 | p < 0.001 |
|  |  | Hemorrhagic | 60.4% (52.7%; 68%) |  |  |
|  | Previous Rankin Scale | 0-2 | 26.5% (24%; 29%) | p < 0.001 | p < 0.001 |
|  |  | 3-5 | 57.6% (53.4%; 61.8%) |  |  |
|  | Male gender | No | 39% (36%; 42%) | 0.007 | 0.011 |
|  |  | Yes | 32.6% (29.1%; 36%) |  |  |
|  | Hypertension | No | 33.5% (28%; 39%) | 0.253 | 0.268 |
|  |  | Yes | 36.9% (34.4%; 39.4%) |  |  |
|  | Diabetes mellitus | No | 34.8% (32%; 37.6%) | 0.093 | 0.120 |
|  |  | Yes | 39.4% (35.4%; 43.3%) |  |  |
|  | Dyslipidemia | No | 39.5% (36.3%; 42.7%) | 0.006 | 0.010 |
|  |  | Yes | 32.9% (29.7%; 36.1%) |  |  |
|  | Active smoker | No | 37.2% (34.8%; 39.6%) | 0.030 | 0.046 |
|  |  | Yes | 28.8% (22%; 35.5%) |  |  |
|  | Alcohol overuse | No | 36.8% (34.4%; 39.2%) | 0.159 | 0.191 |
|  |  | Yes | 30.4% (22.1%; 38.8%) |  |  |
|  | Concomitant causes (CSE) | No | 38% (35.5%; 40.5%) | p < 0.001 | p < 0.001 |
|  |  | Yes | 27.6% (22%; 33.1%) |  |  |
|  | Heart failure | No | 34.3% (31.7%; 36.9%) | 0.001 | 0.002 |
|  |  | Yes | 42.8% (38.1%; 47.6%) |  |  |
|  | Coronary disease | No | 36.4% (34%; 38.9%) | 0.953 | 0.953 |
|  |  | Yes | 36.1% (30.2%; 42%) |  |  |
|  | Peripheral vasculopathy | No | 35.9% (33.5%; 38.3%) | 0.173 | 0.195 |
|  |  | Yes | 41.5% (33.4%; 49.6%) |  |  |
|  | Valvulopathy | No | 37.4% (35%; 39.9%) | 0.049 | 0.068 |
|  |  | Yes | 28.4% (22.2%; 34.7%) |  |  |
|  | Anticoagulation therapy | No | 67.1% (63.6%; 70.7%) | p < 0.001 | p < 0.001 |
|  |  | Yes | 16.5% (14.2%; 18.8%) |  |  |
|  | Anticoagulants type | No anticoagulation | 67.1% (63.6%; 70.7%) | p < 0.001 | p < 0.001 |
|  |  | Vitamin K antagonist (VKA) | 20.3% (16.5%; 24.1%) |  |  |
|  |  | Direct oral anticoagulant (DOAC) | 13.7% (10.9%; 16.5%) |  |  |
|  | AF detection timing | known before stroke | 39.1% (36.3%; 41.8%) | p < 0.001 | p < 0.001 |
|  |  | <1 Month | 32.7% (28.3%; 37%) |  |  |
|  |  | 30-90 Days | 7.5% (0%; 15.7%) |  |  |
|  |  | >90 Days | 11.8% (0%; 27.7%) |  |  |
|  | AF monitoring duration (post-discharge) | Known at Hospital Discharge | 38% (35.6%; 40.4%) | p < 0.001 | p < 0.001 |
|  |  | Short | 11.5% (0.7%; 22.2%) |  |  |
|  |  | Intermediate | 3.6% (0%; 10.6%) |  |  |
|  |  | Extended | 9.7% (0%; 20.3%) |  |  |

**Legend:** This table summarizes the unadjusted associations between baseline variables and two outcomes occurring within six months after stroke: (1) early recurrence (ischemic or hemorrhagic), and (2) death as a competing event. For continuous variables, hazard ratios (HR) or subdistribution hazard ratios (sHR) with 95% confidence intervals (CI) are shown. For categorical variables, recurrence and mortality rates (with 95% CI) are presented by group. P-values and Q-values (adjusted for multiple comparisons using the false discovery rate method) are included. These results informed variable selection for multivariable models.

**Abbreviations:** AC = anticoagulation; AF = atrial fibrillation; CI = confidence interval; CSE = concomitant stroke etiology; DOAC = direct oral anticoagulant; ER = early recurrence; HR = hazard ratio; ICH = intracerebral hemorrhage; NIHSS = National Institutes of Health Stroke Scale; Q = false discovery rate-adjusted p-value; sHR = subdistribution hazard ratio; TIA = transient ischemic attack; VKA = vitamin K antagonist.

**Supplementary Table S2. Fine-Gray Competing Risk Model for Ischemic Recurrence in Patients with Ischemic Stroke and Atrial Fibrillation**

| Variable | sHR (95% CI) | p-value |
| --- | --- | --- |
| Initial stroke severity (NIHSS) | 0.919 (0.888–0.952) | < 0.001 |
| CHA₂DS₂-VASc score | 1.294 (1.054–1.588) | 0.014 |
| Active smoker | 1.179 (0.6–2.315) | 0.633 |
| Coronary artery disease | 0.995 (0.566–1.749) | 0.987 |
| Heart failure | 1.041 (0.613–1.77) | 0.881 |
| Valvulopathy | 0.977 (0.536–1.78) | 0.94 |
| Concomitant stroke etiology | 1.979 (1.27–3.084) | 0.003 |
| AF Monitoring Duration: short (≤3 days) | 2.264 (0.804–6.371) | 0.122 |
| AF Monitoring Duration: intermediate (>3 to <14 days) | 0.678 (0.088–5.249) | 0.71 |
| AF Monitoring Duration: prolonged (≥14 days) | 3.357 (1.156–9.746) | 0.026 |
| Interaction: AC + Known AF (ref) | 0.676 (0.383–1.193) | 0.176 |
| Interaction: No AC + AF <1 month | 2.236 (1.123–4.45) | 0.022 |
| Interaction: AC + AF <1 month | 0.796 (0.381–1.66) | 0.542 |
| Interaction: No AC + AF 30–90 days | 4.763 (1.101–20.593) | 0.037 |
| Interaction: AC + AF 30–90 days | 0.863 (0.23–3.235) | 0.827 |
| Interaction: No AC + AF >90 days | 0 (0–0.001) | < 0.001 |
| Interaction: AC + AF >90 days | 0.517 (0.053–5.053) | 0.57 |

**Legend:** This table presents the results of a multivariable Fine-Gray competing risk model restricted to patients with ischemic stroke as the index event. Only ischemic stroke recurrence was considered as the primary outcome, while death was treated as a competing event. The model includes baseline clinical predictors, AF monitoring duration after hospital discharge, and an interaction term between anticoagulation therapy (AC) and timing of AF detection. Subdistribution hazard ratios (sHR) with 95% confidence intervals (CI) and p-values are reported. The reference group for the interaction was anticoagulated patients with AF diagnosed before hospital discharge. **Abbreviations:** AC = anticoagulation; AF = atrial fibrillation; CI = confidence interval; CSE = concomitant stroke etiology; DOAC = direct oral anticoagulant; NIHSS = National Institutes of Health Stroke Scale; sHR = subdistribution hazard ratio; VKA = vitamin K antagonist.

# **Supplementary Table S3.** **Comparison of CHA₂DS₂-VASc and CHADS-VA Scores in Multivariable Models**

| **Risk Score** | **Model** | **Estimate (95% CI)** | **p-value** | **C-index** | **AIC** |
| --- | --- | --- | --- | --- | --- |
| CHA₂DS₂-VASc | Cox | HR = 1.28 (1.05–1.55) | 0.0128 | 0.711 | 1509.3 |
| CHADS-VA | Cox | HR = 1.26 (1.06–1.50) | 0.013 | 0.707 | 1509.5 |
| CHA₂DS₂-VASc | Fine-Gray | sHR = 1.252 (1.031–1.521) | 0.023 | – | – |
| CHADS-VA | Fine-Gray | sHR = 1.27 (1.001–1.612) | 0.049 | – | – |

**Legend:** This table summarizes the results of multivariable Cox and Fine-Gray models comparing the predictive performance of the CHA₂DS₂-VASc and CHADS_VA scores. Both models included the same set of covariates. For Cox models, discrimination was assessed using Harrell’s concordance index (C-index) and model fit using the Akaike Information Criterion (AIC). Hazard ratios (HR) and subdistribution hazard ratios (sHR) are shown with 95% confidence intervals (CI).

**Supplementary Table S4.** Fine-Gray competing-risk model for early stroke recurrence with anticoagulation therapy as a time-dependent covariate.

| Variable | sHR (95% CI) | p-value |
| --- | --- | --- |
| CHA₂DS₂-VASc score | 1.346 (1.1–1.646) | 0.004 |
| Initial stroke severity (NIHSS) | 0.962 (0.931–0.994) | 0.02 |
| Active smoker | 1.047 (0.526–2.087) | 0.895 |
| Coronary artery disease | 0.826 (0.466–1.465) | 0.514 |
| Heart failure | 0.963 (0.557–1.664) | 0.892 |
| Valvulopathy | 1.163 (0.65–2.081) | 0.611 |
| Concomitant stroke etiology | 2.213 (1.431–3.424) | < 0.001 |
| AF detection timing: <1 month | 1.639 (1.034–2.598) | 0.035 |
| AF detection timing: 30–90 days | 1.695 (0.525–5.469) | 0.378 |
| AF detection timing: >90 days | 0.356 (0.039–3.28) | 0.362 |
| AF Monitoring Duration: short (≤3 days) | 2.281 (0.849–6.131) | 0.102 |
| AF Monitoring Duration: intermediate (>3 to <14 days) | 0.385 (0.05–2.98) | 0.361 |
| AF Monitoring Duration: prolonged (≥14 days) | 2.312 (0.752–7.107) | 0.144 |
| Anticoagulation therapy (time-dependent) | 0.371 (0.249–0.551) | < 0.001 |

**Legend:** This table presents the results of a multivariable Fine-Gray competing-risk model for early stroke recurrence, incorporating anticoagulation therapy as a time-dependent covariate. The model adjusts for baseline clinical predictors, including CHA₂DS₂-VASc score, initial stroke severity (NIHSS=National Institutes of Health Stroke Scale), concomitant stroke etiology, AF detection timing, and monitoring duration after discharge. Anticoagulant type was not included in this model, as the timing of anticoagulation exposure was modeled dynamically. Subdistribution hazard ratios (sHR), 95% confidence intervals (CI), and p-values are reported for each covariate.

**Supplementary Figure 1. Recurrence rates prior to atrial fibrillation detection by timing group**


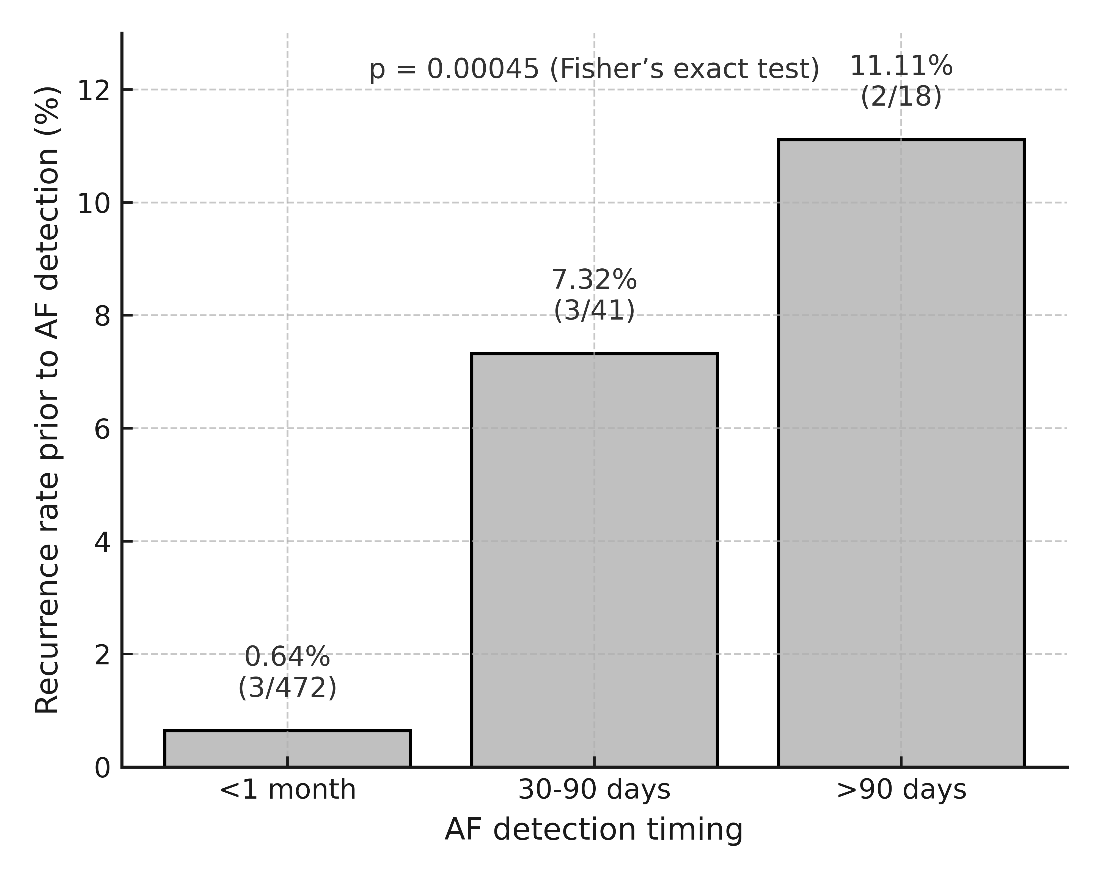


**Legend:** Recurrence rates prior to atrial fibrillation (AF) detection across timing groups in patients with AF detected after hospital admission. Bars represent the proportion of patients who experienced early recurrence before AF detection in each group (<1 month, 30–90 days, >90 days). Absolute numbers (n/N) and proportions are displayed above each bar. Group comparisons were performed using Fisher’s exact test (p = 0.00045).

**Supplementary Figure 2. Interaction between anticoagulation and timing of AF detection on early stroke recurrence**


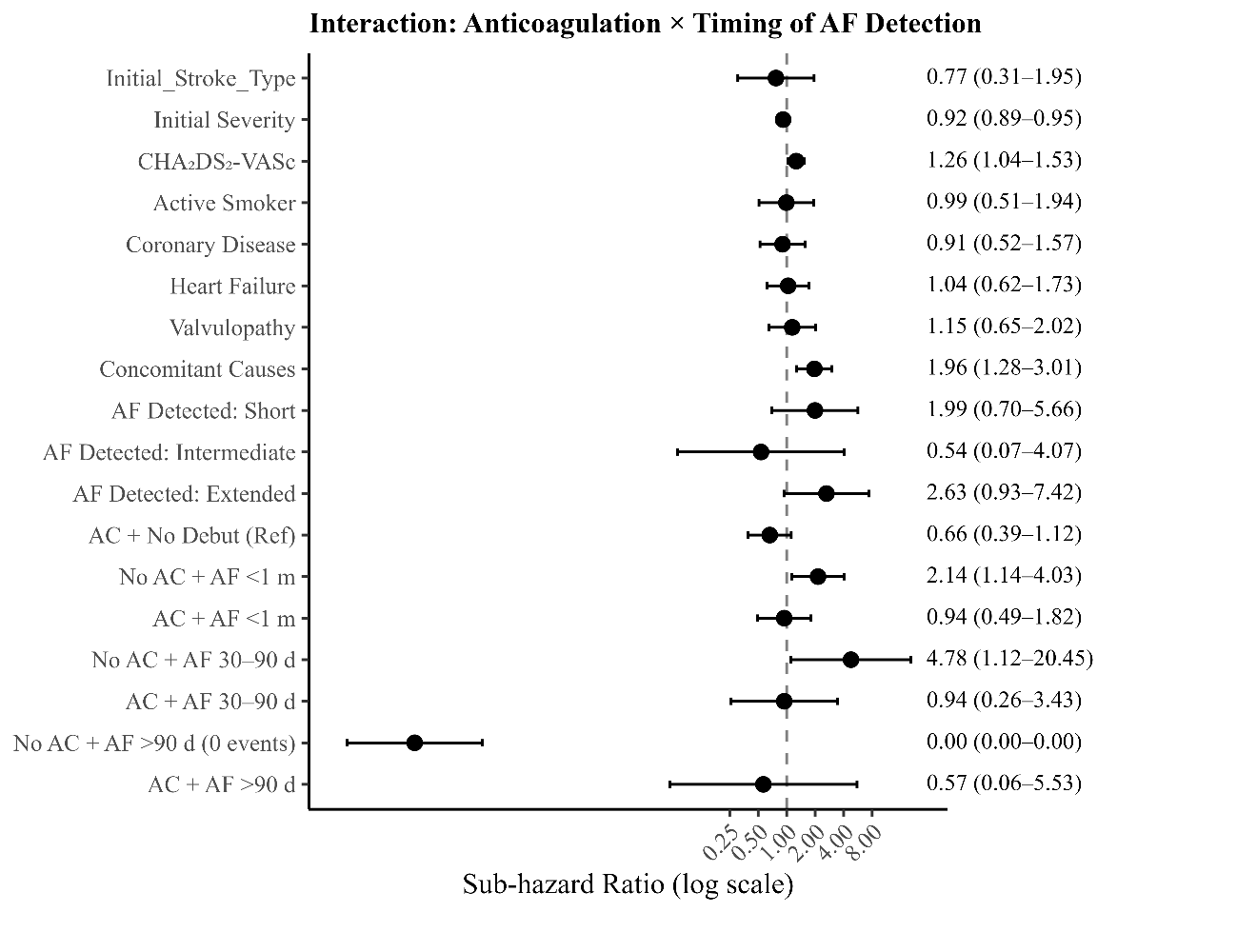


**Legend:** Subdistribution hazard ratios (sHR) and 95% confidence intervals from a Fine-Gray competing-risk model including an interaction term between oral anticoagulation (AC) and timing of AF detection (<1 month, 30–90 days, >90 days). The reference group is “AC + known AF before stroke.” No recurrences occurred in non-anticoagulated patients with AF detected >90 days, hence this combination is not shown on the logarithmic scale. The model was adjusted for CHA₂DS₂-VASc, NIHSS, concomitant stroke etiology, and other covariates reported in Table 3.
